# Uncovering hypothalamic network disruption in ALS

**DOI:** 10.1007/s00415-025-13574-3

**Published:** 2025-12-22

**Authors:** Fabiola Freri, Edoardo Gioele Spinelli, Elisa Canu, Silvia Basaia, Veronica Castelnovo, Hans-Peter Müller, Jan Kassubek, Albert C. Ludolph, Sruthi Sankari Krishnamurthy, Francesco Roselli, Massimo Filippi, Federica Agosta

**Affiliations:** 1 Neuroimaging Research Unit, Division of Neuroscience and Unit of Neurology, IRCCS San Raffaele Scientific Institute, Via Olgettina, 60, 20132 Milan, Italy; 2 Neurology Unit, IRCCS San Raffaele Scientific Institute, Milan, Italy; 3 Vita-Salute San Raffaele University, Milan, Italy; 4 Department of Neurology, University of Ulm, Ulm, Germany; 5 German Center for Neurodegenerative Diseases (DZNE), Ulm, Germany; 6 Neurophysiology Service, IRCCS San Raffaele Scientific Institute, Milan, Italy; 7 Neurorehabilitation Unit, IRCCS San Raffaele Scientific Institute, Milan, Italy

**Keywords:** Amyotrophic lateral sclerosis, Functional connectivity, Hypothalamus, MRI, Hypermetabolism

## Abstract

**Background:**

Structural MRI studies have shown hypothalamic atrophy and altered white matter (WM) connectivity in amyotrophic lateral sclerosis (ALS), as a possible substrate of hypermetabolism in this condition. However, hypothalamic functional connectivity and its association with clinical features in ALS remain unclear. This study explored hypothalamic resting-state functional connectivity (RS-FC) in ALS patients compared to controls and its relationship with disease severity defined by the ALS Functional Rating Scale (ALSFRS-r), body mass index (BMI), disease duration, progression rate, survival, hypothalamic volume, and WM integrity.

**Methods:**

Seventy-one ALS patients and 39 healthy controls underwent structural and RS functional MRI. The bilateral hypothalamus was segmented, and a seed-based RS-FC analysis was performed. Group differences in hypothalamic RS-FC and their correlations with ALSFRS-r scores, BMI, disease duration, progression rate, survival, hypothalamic volume, and WM integrity were assessed. Tract-based spatial statistics was performed to estimate the correlation between WM damage in ALS and hypothalamic RS-FC.

**Results:**

ALS patients showed increased hypothalamic RS-FC with caudate nuclei compared to controls. Additionally, greater disease severity correlated with increased hypothalamic RS-FC with the caudate nuclei and orbitofrontal cortex. Hypothalamic RS-FC mean values also associated with FA in the genu of corpus callosum and forceps minor and disease progression rate. No significant correlations were observed with other clinical features.

**Conclusions:**

These findings support hypothalamic alterations in ALS. Early detection of hypothalamic changes could be useful in prognostic stratification and evaluating intervention effects.

## Introduction

Amyotrophic lateral sclerosis (ALS) is a neurodegenerative disorder characterized by the progressive degeneration of upper motor neurons in the motor cortex and lower motor neurons in the spinal cord. This degeneration leads to muscle weakness [1], which typically begins asymmetrically in the limbs [2, 3], and/or bulbar dysfunction. ALS typically manifests between the ages of 40 and 60, with a median survival time of 2 to 5 years following onset, with respiratory failure as the most common cause of death [4].

While ALS is traditionally considered a motor system disease, it is increasingly recognized as a condition that also impacts cognitive functioning and behaviour [5], but also metabolic processes, sleep, and autonomic regulation [6–9]. These non-motor alterations significantly affect patient well-being and survival. Among these, hypermetabolism has recently been identified as a clinical feature of ALS [10, 11], leading to weight loss and a reduced body mass index (BMI), both of which are strongly associated with higher mortality rates.

Histopathological investigations have shown that the hypothalamus is directly affected in ALS, with post-mortem analyses revealing the presence of TDP-43 protein aggregates within this region [11, 12]. Notably, TDP-43 involvement in the lateral hypothalamus has been linked to reduced BMI in affected individuals [12]. As a central regulator of energy balance, the hypothalamus maintains homeostasis by integrating peripheral signals—such as leptin, a hormone correlated with body fat mass and inversely associated with ALS risk [13]—to modulate food intake and energy expenditure [14]. Therefore, hypothalamic dysfunction could explain the metabolic alterations observed in ALS, such as hypermetabolism and weight loss [15].

Neuroimaging studies support this view, demonstrating a reduction in hypothalamic volume in ALS patients compared to healthy controls [16, 17], with greater atrophy correlating with accelerated weight loss, lower BMI, and poorer survival in ALS cases [10, 17–19]. These alterations may reflect impaired satiety signalling, dysregulated energy expenditure, or broader hormonal dysfunction. Although loss of appetite in ALS is likely multifactorial, increasing data suggest that disrupted hypothalamic circuits, rather than solely dysphagia, psychosocial distress, depression, physical disability, contribute to reduced food intake. Imaging and post-mortem studies point toward the involvement of neuropeptidergic neurons such as POMC, MCH, and orexin, whose impairment may exacerbate nutritional decline [15, 19].

More broadly, the hypothalamus is emerging as a critical hub implicated not only in metabolic alterations but also in sleep regulation, cognition, and behaviour—domains increasingly recognised as clinically significant in ALS. In line with this, alterations in white matter (WM) structural connectivity between the hypothalamus and other non-motor brain regions such as the orbitofrontal and insular regions have been recently reported [16].

While structural changes in the hypothalamus have been documented, evidence regarding its functional connectivity (FC) in ALS remains limited. To date, only one study has reported a significant increase in hypothalamic resting state FC (RS-FC) in ALS patients compared to controls, mainly in the left superior and middle temporal gyrus, right inferior frontal gyrus, right putamen, and left precuneus [20]. However, the relationship between hypothalamic RS-FC, structural WM changes, and clinical outcomes, such as BMI and disease severity, remains unclear.

In this study, we investigated RS-FC of the hypothalamus in ALS patients compared to healthy controls. Furthermore, we wished to examine the relationship between hypothalamic RS-FC, WM structural changes, and clinical features to better understand the hypothalamic contribution to ALS pathophysiology.

## Methods

### Participants

In this study, we included 71 patients with a clinical diagnosis of probable or definite ALS, according to the El Escorial Criteria [1], from those attending the Center for Neurodegenerative Diseases-DZNE, Ulm, Germany, between 2013 and 2014. Thirty-nine age- and sex- matched healthy subjects were also recruited among non-consanguineous relatives and by word of mouth. We included only subjects who underwent a clinical evaluation and a structural and RS functional MRI scan. All participants were excluded if they had any history of other neurological or psychiatric disorders and other causes of focal or diffuse brain damage, including lacunae and extensive cerebrovascular disorders on conventional MRI scans.

### Clinical assessment

At study enrolment, disease severity was scored using the revised ALS Functional Rating Scale (ALSFRS-r) [21], and BMI was recorded for patients. Disease duration was calculated from symptom onset to MRI in months. Disease progression rate was defined as the difference between the maximum ALSFRS-r score (i.e. 48) and the patient’s ALSFRS-r score at the time of the MRI scan, divided by the patient’s disease duration. If available from clinical charts (*n* = 39), survival time after MRI was also recorded.

### Genetic analysis

Analysis of the *C9ORF72* repeat length was performed by fragment length analysis and repeat-primed PCR [22, 23]. Electrophoresis was performed on an ABI PRISM® 3130 Genetic Analyzer (Life Technologies, Foster City, California, USA). The data were analysed using the Peak Scanner software (Applied Biosystems, Waltham, Massachusetts, USA). Samples with a sawtooth pattern in the repeat-primed PCR were further analysed using Southern blot. Screening for *SOD1* was done by Sanger sequencing for all coding exons and flanking 50 bps of *SOD1*.

### MRI acquisition

MRI scans were obtained using a 3.0 T scanner (Allegra Siemens Medical, Erlangen, Germany) with a T1-weighted magnetisation-prepared gradient echo image (MPRAGE) sequence (TR = 2200 ms, TE = 4.7 ms; 192 contiguous sagittal slices with voxel size = 1.0 × 1.1 × 1.0 mm^3^, matrix size = 256 × 192 × 256). Human whole-brain echo planar images (EPI) were acquired using a blood oxygen level dependent (BOLD) sensitized resting-state fMRI (RS-fMRI) sequence (TR = 2000 ms, TE = 30 ms, flip angle = 90 degrees; FOV = 192 × 192 × 149 mm^3^, matrix size = 64 × 64 × 30, 300 volumes of 30 contiguous transversal slices). The DTI study protocol consisted of 49 gradient directions, including one b0 gradient direction (no gap, 2.2 mm^3^ isovoxels, 96 × 128 × 52 matrix, TE = 85 ms, TR = 7600 ms, *b* = 1000 s/mm^2^).

### MRI analysis

The hypothalamic regions were manually delineated on T1-weighted sequences by two expert raters at the German Center for Neurodegenerative Diseases-DZNE in Ulm, Germany, as previously described [17]. The remaining MRI analyses were performed at the Neuroimaging Research Unit, IRCCS Scientific Institute San Raffaele, Milan, Italy.

#### Hypothalamic segmentation

The MPRAGE images were used for manual delineation of the hypothalamus in the coronal plane using a previously reported landmark-based procedure (Tensor Imaging and Fibre Tracking software, TIFT) [17]. Briefly, a three-step processing pipeline was used: (1) rigid brain normalisation along the anterior commissure (AC)—posterior commissure (PC) axis; (2) spatial upsampling into a study-specific grid (in-plane resolution 62.5 × 62.5 μm^2^, coronal slice thickness 0.5 mm) to improve the accuracy in identifying landmarks and hypothalamic borders; and (3) delineation of the left and right hemispheric hypothalamus by intensity-threshold-based, semi-manual slice-wise identification of the hypothalamus in coronal slices.

#### Resting-state fMRI preprocessing

RS fMRI data processing was performed using the FMRIB software library (FSLv5.0) as outlined previously [24]. The first four volumes of the RS-fMRI data were removed to reach complete magnet signal stabilization. The following FSL-standard preprocessing pipeline was implemented: (1) motion correction using MCFLIRT; (2) high-pass temporal filtering (lower frequency: 0.01 Hz); (3) spatial smoothing (Gaussian Kernel of FWHM 6 mm); (4) automatic removal of motion artifacts using single-session independent component analysis (ICA-AROMA) [25] in order to identify those independent components (ICs) representing motion-related artifacts.

#### Seed-based resting-state functional connectivity

**Fig. 1:**
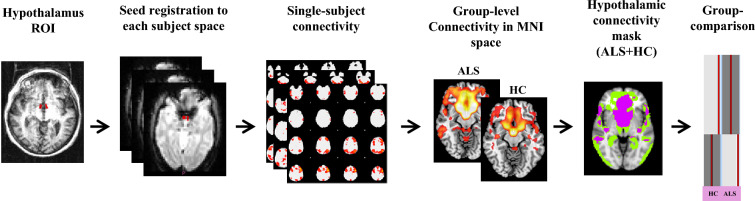
Schematic representation of the procedure for the seed-based resting state functional connectivity. The seed region of interest (ROI) was manually defined in subject's native T1-weighted space. The mean time-series were extracted, and subject-level maps of all positively predicted voxels for each regressor were obtained. Subject-level maps were finally registered to the MNI standard template for the statistical analysis. Here we provided an illustrative example of analysis: seed-based functional connectivity was compared between the patient group and the matched group of controls using a general linear model which includes group as independent factor. *ALS* amyotrophic lateral sclerosis, *HC* healthy controls, *ROI* region of interest

Bilateral hypothalamus was selected as the seed region for the analysis. Seed-based RS-FC was performed using a two-step regression analysis as implemented in the FMRIB Software Library (FSLv5). First, time series of WM, cerebrospinal fluid (CSF), and whole brain volumes in RS fMRI native space were extracted from the preprocessed and denoised data, and their effects were regressed out using the FMRI Expert Analysis Tool (FEAT). Subsequently, seed mean time series were then calculated. This step generated subject-level maps of positively predicted voxels for each regressor. Subject-level maps were registered to the MNI standard template for the statistical analysis (Fig. 1).

#### DTI preprocessing

DT MRI analysis was performed using FMRIB software library (FSL, version 5.0.9) tools (http://www.fmrib.ox.ac.uk/fsl/fdt/index.html) and Jim.

The diffusion-weighted (DW) data were skull-stripped using the Brain Extraction Tool (BET) implemented in FSL. Diffusion-weighted images were corrected for distortions caused by eddy currents and for head movements, using Jim8 Software (Jim 8.0, Xinapse Systems Ltd, Colchester, UK, http://www.xinapse.com). This eddy-current/motion correction step combines a rigid-body 3D motion correction (6 parameters) with a constrained non-linear warping (8 parameters) based on a model of the expected eddy-current distortions. The DT was estimated on a voxel-by-voxel basis using the DTIfit toolbox, part of the FMRIB Diffusion Toolbox within FSL, and mean diffusivity (MD) and fractional anisotropy (FA) maps were computed. Maps of axial diffusivity (axD), which are equivalent to the magnitude of the largest eigenvalue of the tensor, and radial diffusivity (radD), which is the average of the two smallest eigenvalues of the tensor, were also calculated.

#### Tract-Based Spatial Statistics (TBSS)

Tract-based spatial statistics (TBSS) version 1.2 (http://www.fmrib.ox.ac.uk/fsl/tbss/index.html) was used to perform the multi-subject diffusion tensor MRI (DT-MRI) analysis. FA volumes were aligned to a target image using the following procedure: (1) the FA template in standard space (provided by FSL) was selected as the target image, (2) the non-linear transformation that mapped each subject’s FA to the target image was computed using the FMRIB’s Non-linear Image Registration Tool (FNIRT), and (3) the same transformation was used to align each subject’s FA to the standard space. A mean FA image was then created by averaging the aligned individual FA images and thinned to create an FA skeleton representing WM tracts common to all subjects. The FA skeleton was thresholded at a value of 0.2 to exclude voxels with low FA values, which are likely to include grey matter or cerebrospinal fluid. Individual maps of MD, FA, axD, and radD were projected onto this common skeleton.

#### Voxel-Based Morphometry (VBM)

Voxel-based morphometry (VBM) was performed using SPM12 (fil.ion.ucl.ac.uk/spm/) and diffeomorphic anatomical registration exponentiated lie algebra (DARTEL) registration method [26] to investigate gray matter (GM) volume alterations at a whole-brain level. Details of the VBM pipeline have been described previously [27].

### Statistical analysis

#### Demographic and clinical data

To compare demographic characteristics, clinical features, and hypothalamic volumes between groups, one-way ANOVA was used for continuous variables, and Pearson’s χ^2^ test was applied for categorical variables. To assess potential sexual dimorphism, one-way ANOVA was used for comparing male and female ALS patients on clinical variables (disease duration, disease progression rate, ALSFRS-r score, BMI), hypothalamic RS-FC mean values, and hypothalamic volume. All analyses were performed with age as a covariate and thresholded at *p* < 0.05. The statistical analyses were performed with SPSS software (version 25.0; IBM Corp., Armonk, NY, USA).

#### Seed-based resting-state functional connectivity

Mean hypothalamic RS-FC was obtained for each group and was compared between ALS patients and healthy controls using GLMs, which included RS-FC maps as dependent variables, age as a covariate, and the combined mean RS-FC of ALS and control groups as a mask. For the correlation analysis between RS-FC changes and clinical features, we used GLMs, which included RS-FC maps as dependent variables, ALSFRS-r, disease duration, disease progression rate, survival, hypothalamic volumes, or BMI as independent variables, age as a covariate, and the mean RS-FC of the ALS group as a mask.

For all analyses, corrections for multiple comparisons were carried out at a cluster level using Gaussian random field theory, *z* > 2.3; cluster significance: *p* < 0.05, corrected for multiple comparisons.

#### TBSS: between-group comparisons and correlations

To describe WM disruption in ALS patients, a TBSS analysis was performed using a permutation-based inference tool for nonparametric statistical thresholding (“randomise”, FSL [28]). The number of permutations was set at 5000 [28]. First, we compared DT MRI parameters between groups using GLMs that included skeletonized MD, FA, axD, and radD maps, separately, as dependent variables and age as a covariate. Second, to investigate whether the DT MRI abnormalities were related to hypothalamic RS-FC changes in ALS, we performed correlations using GLMs. Each GLM included the skeletonized DT MRI maps, separately, as dependent variables and the RS-FC mean values, extracted for each subject from the significant cluster identified in the seed-based comparison between groups, as independent variables. TBSS analyses were thresholded at *p* < 0.05, corrected for multiple comparisons at the cluster level using the threshold-free cluster enhancement (TFCE) option.

#### Correlation analysis

Correlations between clinical features of ALS patients (i.e. ALSFRS-r score, disease duration, disease progression rate, survival time, hypothalamic volumes, and BMI) and the RS-FC mean values were tested using the Pearson coefficient for the normally distributed variables, and the Spearman coefficient was used for non-normally distributed variables. Analyses were performed with age as a covariate and thresholded at *p* < 0.05. The statistical analyses were performed with SPSS software (version 25.0; IBM Corp., Armonk, NY, USA).

#### Survival analysis

Survival analysis was performed using Kaplan–Meier curves and the log-rank test. For individuals without a documented date of death, we used the last censoring date when patients were known to be alive. Patients were dichotomized according to the median RS-FC value to compare survival probability between “low” and “high” RS-FC groups.

#### VBM: between-group comparisons

VBM group comparisons were tested using ANOVA models in SPM12, adjusting for age. Results were assessed at *p* < 0.05, family-wise error–corrected for multiple comparisons.

## Results

### Clinical, genetic, and volumetric data

Table 1 summarizes the sociodemographic, clinical, and hypothalamic volumetric features of the sample. Patients and healthy controls were comparable for age and sex. Five patients with ALS-related pathogenic variants were identified (4 *C9ORF72* and 1 *SOD1*). The normalized hypothalamic volume was significantly lower in ALS patients than in controls.

**Table 1:** Demographic and clinical features of ALS patients and healthy controls

	HCs	ALS patients	*p* value
*N*	39	71	–
Age [years]	54.32 ± 12.28 (22–76)	58.28 ± 13.91 (20–85)	0.14
Sex [women/men]	18/21	29/42	0.59
ALSFRS-r	–	4.5 ± 5.3 (23–48)	–
Disease duration [months]		16.7 ± 13.8 (1–60)	–
Normalized hypothalamic volume [mm^3^]	0.93 ± 0.12 (0.62–1.12)	0.81 ± 0.14 (0.50–1.21)	< 0.001

Values are means ± standard deviations (min – max) or frequencies

*ALS* amyotrophic lateral sclerosis, *ALSFRS-r* Amyotrophic Lateral Sclerosis Functional Rating Scale revised, *HC* Healthy controls, *N* number

No significant sex-related differences were found in disease duration, disease progression rate, ALSFRS-R score, or BMI when controlling for age. Similarly, age-adjusted hypothalamic RS-FC mean values and hypothalamic volume did not differ between male and female ALS patients.

### Seed-based resting-state functional connectivity

**Fig. 2:**
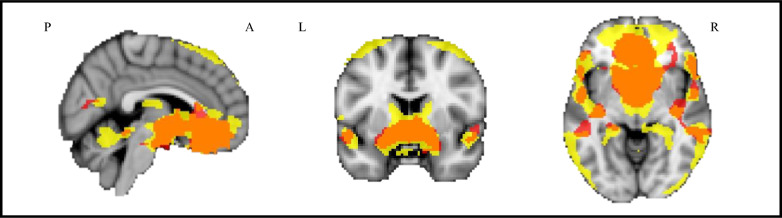
Mean resting-state functional connectivity between hypothalamus and the rest of the brain. HC (red colour), ALS patients (yellow colour) and their overlap (orange colour) are shown. Results are overlaid on the Montreal Neurological Institute (MNI) standard brain and displayed at *p* < 0.05 family-wise error (FWE) corrected for multiple comparisons at a cluster level

**Fig. 3:**
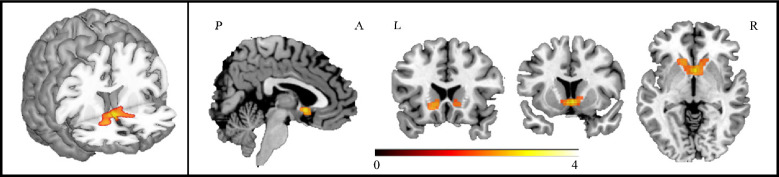
Brain regions where ALS patients showed increased hypothalamic resting-state functional connectivity compared to healthy controls. Results are overlaid on the Montreal Neurological Institute (MNI) standard brain and displayed at *p* < 0.05 family-wise error (FWE) corrected for multiple comparisons at a cluster level. Colour bar represents *Z* values

**Fig. 4:**
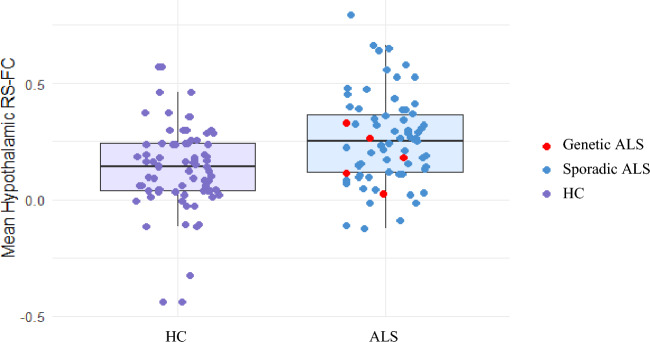
Box plots of the mean increased hypothalamic–caudate connectivity in healthy controls and ALS. Genetic ALS cases are reported as red dots

For each group, Fig. 2 reports the mean RS-FC connectivity map between the bilateral hypothalamus and the rest of the brain. In healthy controls, hypothalamic RS-FC was primarily observed with the putamen, orbitofrontal regions, secondary and primary visual areas, and the medial temporal gyrus. In ALS patients, the connectivity map was more distributed, extending to the supplementary motor area (SMA) and the superior medial frontal area. Compared to controls, ALS patients showed increased RS-FC between the bilateral hypothalamus and the bilateral caudate nucleus (Fig. 3, Table 2). The individual mean hypothalamic RS-FC values within voxels showing significant differences between groups demonstrated a similar distribution in sporadic ALS patients and those with a pathogenic genetic mutation (Fig. 4).

**Table 2:** Increased hypothalamic resting state functional connectivity in ALS compared to HC

	Brain region	*N* of voxels	Z	x	y	z
ALS > HC	Right Caudate nucleus	442	3,94	2	10	− 6
	Left Caudate nucleus		3,17	− 12	22	− 6

Coordinates (x, y, z) are in Montreal Neurological Institute (MNI) space. Results are displayed at *p* < 0.05 family-wise error (FWE) corrected for multiple comparisons at a cluster level

*ALS* amyotrophic lateral sclerosis, *HC* healthy controls, *RS-FC* resting state functional connectivity, *L* left, *N* number, *R* right

**Fig. 5:**
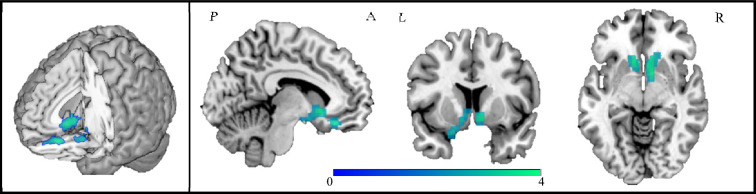
Brain regions where hypothalamic resting state functional connectivity showed a negative correlation with ALSFRS-r scores. Results are overlaid on the Montreal Neurological Institute (MNI) standard brain and displayed at *p* < 0.05 family-wise error (FWE) corrected for multiple comparisons at a cluster level. Colour bar represents *Z* values

At voxel-wise analysis, ALS patients showed a negative correlation between ALSFRS-r scores and hypothalamic RS-FC with bilateral caudate nucleus, orbitofrontal area, left amygdala, and right inferior frontal gyrus pars orbitalis (Fig. 5, Table 3).

**Table 3:** Negative correlation between ALSFRS-r scores and hypothalamic resting state functional connectivity in ALS patients

	Brain region	*N* of voxels	Z	x	y	z
ALSFRS-r *vs* hypothalamic RS-FC	Left Caudate nucleus	573	4,04	− 10	18	− 6
	Left Orbitofrontal area		3,93	− 6	32	− 16
	Left Orbitofrontal area		3,38	− 20	10	− 22
	Left Amygdala		2,96	− 10	4	− 24
	Left Amygdala		2,86	− 20	4	− 24
	Left Orbitofrontal area		2,72	− 4	46	− 14
	Right Caudate nucleus	363	4,1	8	14	− 8
	Right Caudate nucleus		3,98	12	22	− 8
	Right Pars Orbitalis		3,01	16	16	− 26
	Right Orbitofrontal area		2,89	16	16	− 20

Coordinates (x, y, z) are in Montreal Neurological Institute (MNI) space. Results are displayed at *p* < 0.05 family-wise error (FWE) corrected for multiple comparisons at a cluster level

*ALS* amyotrophic lateral sclerosis, *ALSFRS-r* Amyotrophic Lateral Sclerosis Functional Rating Scale revised, *HC* healthy controls, *RS-FC* resting state functional connectivity, *L* left, *N* number, *R* right

No significant correlations were observed between hypothalamic RS-FC and disease duration, survival, hypothalamic volume and BMI at a voxel-wise level. The mean RS-FC values within ALS-affected voxels showed significant correlations with ALSFRS-r scores (*r* = − 0.33, *p* = 0.005) and disease progression rate (*r* = 0.27, *p* < 0.02).

**Fig. 6:**
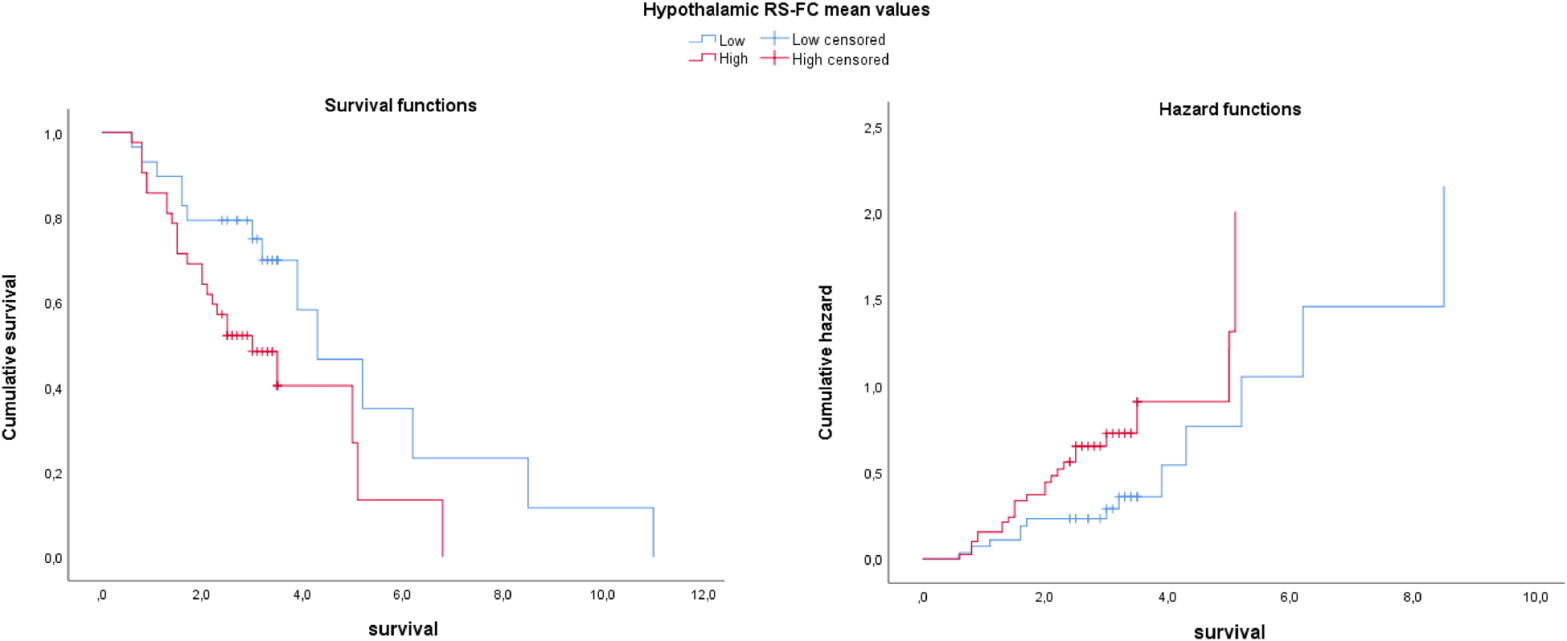
Kaplan–Meier survival and hazard functions for patients stratified according to hypothalamic RS-FC mean values (low RS-FC in blue *vs* high RS-FC in red, median split). Patients with higher hypothalamic RS-FC showed significantly shorter survival compared to those with lower RS-FC (left). The cumulative hazard plot illustrates the increased risk of mortality over time in the high RS-FC group (right)

Kaplan–Meier survival analysis showed that patients with higher hypothalamic RS-FC exhibited significantly decreased survival probability compared to those with lower RS-FC (log-rank *χ*^2^ = 4.75, *p* = 0.03) (Fig. 6). No significant correlations were observed with other clinical features (Table 4).

**Table 4:** Correlations between the hypothalamic RS-FC mean values and clinical features in ALS patients

	*N*	Hypothalamic RS-FC mean values		
		Mean – SD (min -max)	*r*	*p* value
ALSFRS-r	71	40.45 ± 5.32 (23–48)	− 0.33	**0.005**
Disease progression rate	71	0.94 ± 1.28 (0–6.50)	0.27	**0.02**
Survival	39	2.74 ± 2.30 (0.56–10.96)	–	**0.03**
Hypothalamic volume	71	0.81 ± 0.14 (0.50–1.21)	0.15	0.23
BMI	61	25.01 ± 4.04 (15.94–35.43)	0.20	0.12
Disease duration	71	16.66 ± 13.80 (1–60)	− 0.06	0.62

Values are means ± standard deviations (min – max)

*ALSFRS-r* Amyotrophic Lateral Sclerosis Functional Rating Scale revised, *BMI* body mass index, *N* number, *RS-FC* resting-state functional connectivity, *SD* standard deviation

### Tract-Based Spatial Statistics (TBSS)

**Fig. 7:**
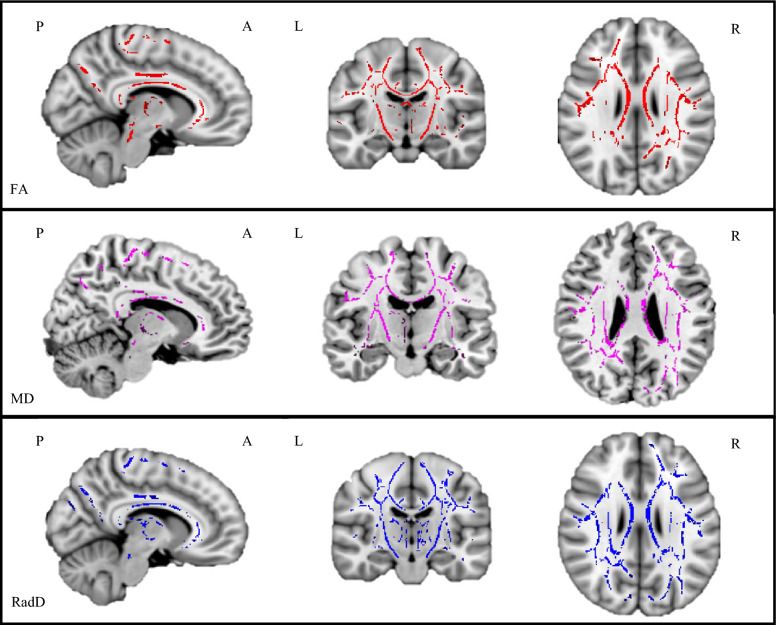
Differences in diffusivity measures between ALS patients and healthy controls. Brain regions showing decreased fractional anisotropy (FA, red), increased mean diffusivity (MD, pink) and increased radial diffusivity (radD, blue) in ALS patients compared to healthy controls. Results are overlaid on the Montreal Neurological Institute (MNI) standard brain in neurological convention (right is right) and displayed at *p* < 0.05 threshold-free cluster enhancement

Compared to healthy controls, ALS patients showed a widespread pattern of decreased FA, along with increased radD and MD, which involved the bilateral corticospinal tract (CST), and the bilateral long intrahemispheric and interhemispheric tracts, in particular the superior longitudinal fasciculus (SLF), and both the body and genu of the corpus callosum (CC) (Fig. 7). No significant differences in axD values were observed between groups.

**Fig. 8:**
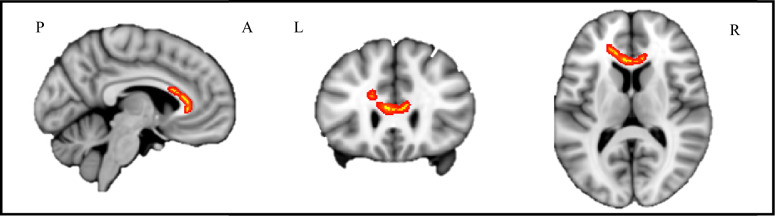
Correlations between fractional anisotropy and mean hypothalamic resting state functional connectivity values. Brain regions showing the negative correlation between fractional anisotropy and mean hypothalamic resting state functional connectivity values extracted from the significant cluster identified in the seed-based group comparison. Results are overlaid on the Montreal Neurological Institute (MNI) standard brain in neurological convention (right is right) and displayed at *p* < 0.05 threshold-free cluster enhancement

In ALS cases we observed a relationship between decreased FA in the genu of the CC and in the forceps minor and higher hypothalamic RS-FC within the significant cluster identified in the seed-based comparison between ALS and controls (Fig. 8). No significant correlations with MD, axD, or radD parameters were observed*.*

### Voxel-Based Morphometry (VBM)

No significant GM differences were observed between ALS and healthy controls.

## Discussion

This study aimed to explore hypothalamic RS-FC in patients with ALS and its association with clinical and structural MRI features. The analysis revealed two main findings. First, in comparison to healthy controls, ALS patients exhibited increased hypothalamic RS-FC with the caudate nuclei. Second, within the ALS patient group, greater disease severity and WM damage were associated with enhanced hypothalamic RS-FC with the bilateral caudate nuclei, orbitofrontal regions, and amygdala. Importantly, these findings were independent of patients’ genetic status and GM atrophy.

The pathological involvement of the hypothalamus in patients with ALS has been demonstrated by autopsy studies showing local pathological accumulation of TDP-43 protein inclusions [11, 12]. Moreover, the presence of TDP-43 pathology in the lateral hypothalamus has been associated with a lower BMI [12]. Recent studies in vivo have demonstrated a significant reduction in total hypothalamic volume in ALS patients compared to controls [16, 17, 29, 30]. Hypothalamic atrophy appears to be associated with lower BMI [16, 17] and is thought to play a role in metabolic processes. Notably, this volume loss occurs prior to the onset of motor symptoms in carriers of pathogenic mutations [17]. Consistent with these findings, and as part of the sample used in a previous study [17], our study confirms the reduction in hypothalamic volume in ALS patients compared to healthy subjects.

Furthermore, this study investigates hypothalamic RS-FC, offering new insights into the dynamic interactions with other brain regions in ALS patients. To our knowledge, only one other study has investigated hypothalamic RS-FC in ALS patients compared to healthy controls, and has reported a significant enhanced hypothalamic RS-FC with the left superior and middle temporal gyrus, right inferior frontal gyrus, right putamen, and left precuneus [20]. In line with these previous findings, compared to controls, we observed an increased hypothalamic RS-FC of ALS patients with the striatum, in particular with the caudate nuclei.

Several other studies assessing brain functional connectivity have reported increased RS-FC in ALS patients, which has been interpreted either as a secondary neuropathological sign or as a compensatory response to early neuropathological changes [31–34]. The findings of this study are more consistent with the first interpretation, as the observed correlations suggest that increased hypothalamic RS-FC is associated with greater disease severity, faster disease progression, shorter survival, and reduced brain structural connectivity. This is consistent with a disconnection hypothesis, which suggests that increased functional connectivity may result from disrupted local inhibitory neuronal circuits and altered cortical excitability [35]. Specifically, we found that increased RS-FC between the hypothalamus and the bilateral caudate nuclei, orbitofrontal cortex, amygdala, and inferior frontal cortex was associated with greater disease severity, as measured by the ALSFRS-r scale. Moreover, increased hypothalamic RS-FC with the caudate nuclei was related to faster disease progression, supporting a maladaptive role of these rearrangements for the disease course in ALS. Finally, we found that patients with increased hypothalamic RS-FC had significantly shorter survival compared to those with lower hypothalamic RS-FC. All these findings suggest that disruption of the hypothalamic network may reflect a broader vulnerability that influences disease severity, progression, and prognosis.

In our study, higher RS-FC between the hypothalamus and the caudate nuclei was also correlated with microstructural damage of WM tracts (i.e. genu of the CC, forceps minor) that project to the prefrontal and orbitofrontal cortices [36]. This is in line with a previous study investigating hypothalamic structural connectivity in ALS, showing alterations in WM pathways linking the hypothalamus to non-motor regions, including the orbitofrontal and insular areas [16].

Taken together, our findings indicate a possible role of a hypothalamic–striato–frontal network for pathogenic modifications in patients with ALS. There is considerable evidence showing that the hypothalamus, particularly its lateral portion, sends direct projections to the striatum [37]. Additionally, the lateral hypothalamus also projects to the midline thalamic nuclei, which in turn establish widespread connections with corticolimbic regions, such as the amygdala and prefrontal cortex, both of which subsequently project to the striatum. All these pathways play a critical role in conveying energy balance information to behaviour by regulating motivational states associated with hunger and satiety. In particular, the caudate nuclei, amygdala, and orbitofrontal cortex are involved in the reward system, playing a crucial role in linking emotions and memory to feeding behaviour [38–41]. Beyond their functional relevance, several structures connected to the hypothalamus have been repeatedly shown to undergo degeneration in ALS. Diffusion MRI studies have revealed microstructural abnormalities in the basal ganglia, thalamus, hippocampus, and amygdala, with significant associations between diffusion metrics, executive dysfunction, disease duration, and disability, indicating that deep nuclei of frontal-subcortical circuits are affected alongside cortical regions [42]. Complementing these findings, volumetric investigations have documented atrophy of the amygdala, caudate nucleus, and the hippocampal-amygdala transition area [43]. A previous study investigating patients with hypothalamic damage due to craniopharyngioma, compared to individuals with non-functional pituitary adenoma, showed reduced activation in the left caudate nucleus when exposed to food-related images in a task-based fMRI paradigm [40]. The reduced activation in the caudate was indicated as a dysfunction in the mechanisms governing satiety and attention to food. This dysfunction was associated with altered processing of food-related reward stimuli, potentially leading to disordered eating patterns, increased hunger, and a diminished sense of fullness [40]. Although the mechanisms remain unclear, we can suspect similar processes occurring in ALS, where the altered hypothalamic connectivity could play a key role in modulating metabolic and behavioural states.

In this study, the altered FC between the hypothalamus and key motor and non-motor circuits in ALS may be associated with changes in hypothalamic regulation of both metabolic and motivational processes. Hypermetabolism is a well-known feature present in a substantial fraction of patients with ALS, which is independent from reduced food intake (e.g. due to dysphagia) and associated with shorter survival [44, 45]. However, we did not observe a correlation between hypothalamic RS-FC and BMI, in contrast with studies showing correlations between hypothalamic atrophy and lower BMI in ALS [10, 12, 17, 18]. We can speculate that atrophy might be more closely related to the downstream effects of hypermetabolism in ALS (i.e. lower BMI), whereas functional rearrangements might reflect more dynamic – possibly earlier – processes, although the cross-sectional design of this study does not allow us to draw conclusions in this regard. Furthermore, longitudinal measurements of body weight or BMI would provide a more accurate assessment of metabolic changes than a single time-point BMI value. However, historical or longitudinal BMI/weight data were not available for our cohort. Another limitation of our study was the lack of specific metabolic measurements, such as calorimetric measures, which could have provided more precise insights into the metabolic alterations associated with hypothalamic activity. Moreover, recent studies in the sleep domain have suggested that cognitive impairment may be related to hypothalamic dysfunction. However, sleep and cognitive data were not available for our cohort, and this should also be acknowledged as a limitation [19]. Additionally, the study lacked information on the behavioural status of our patients. Finally, we did not observe any sex-related differences in either clinical measures or hypothalamic structure and connectivity. Although prior studies have reported sex differences in ALS risk, phenotype, atrophy, and both functional and structural connectivity [46–50], such effects were not detectable in our cohort. Further studies with larger samples are needed to investigate this important issue in greater depth.

This study highlights the possible critical role of the hypothalamus in ALS and its altered functional connectivity with key brain regions involved in homeostasis and reward processing. These alterations appear to be driven by neuropathological processes linked to disease progression and survival. Further research is needed to elucidate the mechanisms underlying these hypothalamic RS-FC changes and their relationship with metabolic alterations, such as hypermetabolism, frequently observed in ALS. Exploring the dynamic interaction between the hypothalamus and other brain regions could provide insights into the non-motor symptoms of ALS, while also identifying potential biomarkers and therapeutic targets for improving disease management.

## Data Availability

The dataset and codes used for this study will be made available by the corresponding author on request.
